# Field template-based design and biological evaluation of new sphingosine kinase 1 inhibitors

**DOI:** 10.1007/s10549-018-4900-1

**Published:** 2018-07-24

**Authors:** Heba Alshaker, Shyam Srivats, Danielle Monteil, Qi Wang, Caroline M. R. Low, Dmitri Pchejetski

**Affiliations:** 1 School of Medicine, University of East Anglia, 2.53 BCRE, Norwich Research Park, Norwich, NR47UQ UK; 2 Department of Pharmacology and Biomedical Sciences, Faculty of Pharmacy and Medical Sciences, University of Petra, Amman, Jordan; 3 Department of Surgery and Cancer, Imperial College London, London, UK; 4 Drug Design Consultant, London, UK

**Keywords:** Sphingosine kinase 1, Molecular field template, Kinase inhibitor, Chemosensitisation, Docetaxel, Breast cancer

## Abstract

**Purpose:**

Sphingosine kinase 1 (SK1) is a protooncogenic enzyme expressed in many human tumours and is associated with chemoresistance and poor prognosis. It is a potent therapy target and its inhibition chemosensitises solid tumours. Despite recent advances in SK1 inhibitors synthesis and validation, their clinical safety and chemosensitising options are not well described. In this study, we have designed, synthesised and tested a new specific SK1 inhibitor with a low toxicity profile.

**Methods:**

Field template molecular modelling was used for compound design. Lead compounds were tested in cell and mouse cancer models.

**Results:**

Field template analysis of three known SK1 inhibitors, SKI-178, 12aa and SK1-I, was performed and compound screening identified six potential new SK1 inhibitors. SK1 activity assays in both cell-free and in vitro settings showed that two compounds were effective SK1 inhibitors. Compound SK-F has potently decreased cancer cell viability in vitro and sensitised mouse breast tumours to docetaxel (DTX) in vivo, without significant whole-body toxicity.

**Conclusion:**

Through field template screening, we have identified a new SK1 inhibitor, SK-F, which demonstrated antitumour activity in vitro and in vivo without overt toxicity when combined with DTX.

**Electronic supplementary material:**

The online version of this article (10.1007/s10549-018-4900-1) contains supplementary material, which is available to authorized users.

## Introduction

Sphingosine kinase 1 (SK1) is a lipid enzyme with oncogenic properties that converts pro-apoptotic lipid second messenger sphingosine into the anti-apoptotic lipid second messenger sphingosine-1-phosphate (S1P). SK1 is tightly regulated by growth factors, cytokines, receptor tyrosine kinases and pro-survival signalling pathways and plays a key role in several fundamental biological processes including cell proliferation, regulation of apoptosis, cell migration, fibrosis, angiogenesis, nociception and inflammatory responses [1–7].

There is compelling evidence that SK1 activation contributes to cancer progression and leads to oncogenic transformation [8], increased tumour growth [9] and impairment of apoptosis [10]. SK1 is a tumour-associated enzyme: high levels of SK1 expression have been shown in various human tumours such as blood, brain, breast, colon, lung, ovary, stomach, uterus, kidney, prostate, rectum and small intestine [11–16] where they enhance tumour neovascularisation [17] and metastatic potential by promoting motility and invasion of cancer cells [18]. High levels of SK1 expression or activity are associated with a poor prognosis in several human cancers, making it a key pathway in the search for targeted therapies [3]. SK1 has an isozyme SK2, which is predominantly localised to cell organelles, and its role in cell proliferation/apoptosis is less well studied.

Multiple SK1 inhibitors have been synthesised and assayed in different biological systems. Sphingosine analogue dimethylsphingosine (DMS), the first direct SK inhibitor, was shown to elicit cancer cell growth inhibition and to provoke apoptosis [19]; however, it lacked specificity affecting multiple lipid and protein kinases and had no selectivity between SK isoforms [20]. Another sphingosine analogue, FTY720, was shown to inhibit SK1 more selectively [21, 22]. It is also a potent apoptosis inducer [23, 24] and has excellent chemo- and radiosensitising properties in prostate and breast cancer models [25–27]. L-threo-dihydrosphingosine (safingol) also demonstrated SK-inhibiting properties [28].

Non-sphingosine analogues F-12509A and B-5354c were synthesised based on extracts from marine fungi *Trichopezizella barbata* [29]. Similar to DMS, F-12509A appears to inhibit SK1 competitively which suggests that the sesquiterpene moiety of F-12509A may mimic the sphingosine-conformation when binding to SK1’s active site, whereas B-5354c demonstrated non-competitive inhibition [29]. The administration of B-5354c triggers dose-dependent apoptosis in LNCaP and PC-3 prostate cancer cells and this may be reversed by upregulation of SK1 [30]. Another group of SK inhibitors (SKI-I-V) also possessing anticancer properties was reported by French et al [14, 31].

A selective SK1 inhibitor (SK1-I) ([(2-hydroxy-1-naphthyl)methylene]-3-(2-napythyl)-1H-pyrazole-5-carbohydrazide) efficiently induces apoptosis in leukaemia cells, but not in normal bone marrow-derived cells [32]. Xiang et al, have developed further SK1-specific inhibitors (6ag, 9ab and 12aa) through a series of modifications of sphingosine [33]. Amidine-based subtype-selective SK1 inhibitors induce reduction of endogenous S1P levels in human leukaemia cells at nanomolar concentrations [34]. A research study investigating the structure–activity relationship of various analogues of SK1-I has demonstrated new inhibitors with optimised selectivity and activity [35], noting that the naphthyl rings were unnecessary for SK1 inhibition. One such discovery was a small molecule SKI-178 which is active both in vitro and in vivo and could be useful in determining the exact functions of SK1 and SK2 isoforms in the development and progression of diseases [35]. Additionally, (S)-FTY720 vinylphosphonate [22] and sphingo-guanidines (LCL146 and LCL351) [36] induce SK1 inhibition in breast and prostate cancer cells and decrease the migration rate of human prostate DU145 cells.

Despite recent advances in SK1 inhibitors synthesis and validation, only most recent ones are isozyme specific (i.e. targeting SK1 and not SK2, which have distinct intracellular functions). Among these, only a few have been studied in animal systems and to our knowledge no general toxicity or side effects studies (such as blood counts, liver and kidney function) have been performed. This is of critical importance, for future drug development as some of better described SK1 inhibitors (e.g. FTY720) have profound side effects that may render them unusable in cancer patients (e.g. lymphopenia, bradycardia and liver function tests derangement) [37, 38]. Finally, we have previously shown SK inhibitor-mediated chemosensitisation to docetaxel (DTX) [6]; however, the clinical safety of such combination was not assessed.

In this study, we have used for the first time field template modelling to design new specific SK1 inhibitors. Lead compounds were tested in cell and mouse cancer models and we found that compound SK-F has potently decreased cancer cell viability in vitro and sensitised mouse breast tumours to DTX *in vivo*, without significant whole-body toxicity.

## Materials and methods

### Reagents

Silica gel 60 high-performance TLC plates were purchased from VWR (West Chester, PA, USA), and [γ-^32^P]-ATP was purchased from Perkin-Elmer (Waltham, MA, USA). SK inhibitor SKI-II was purchased from Selleckchem (Newmarket, UK). All other chemicals were from Sigma Aldrich (Poole, UK).

### Field template molecular modelling

**Fig. 1:**
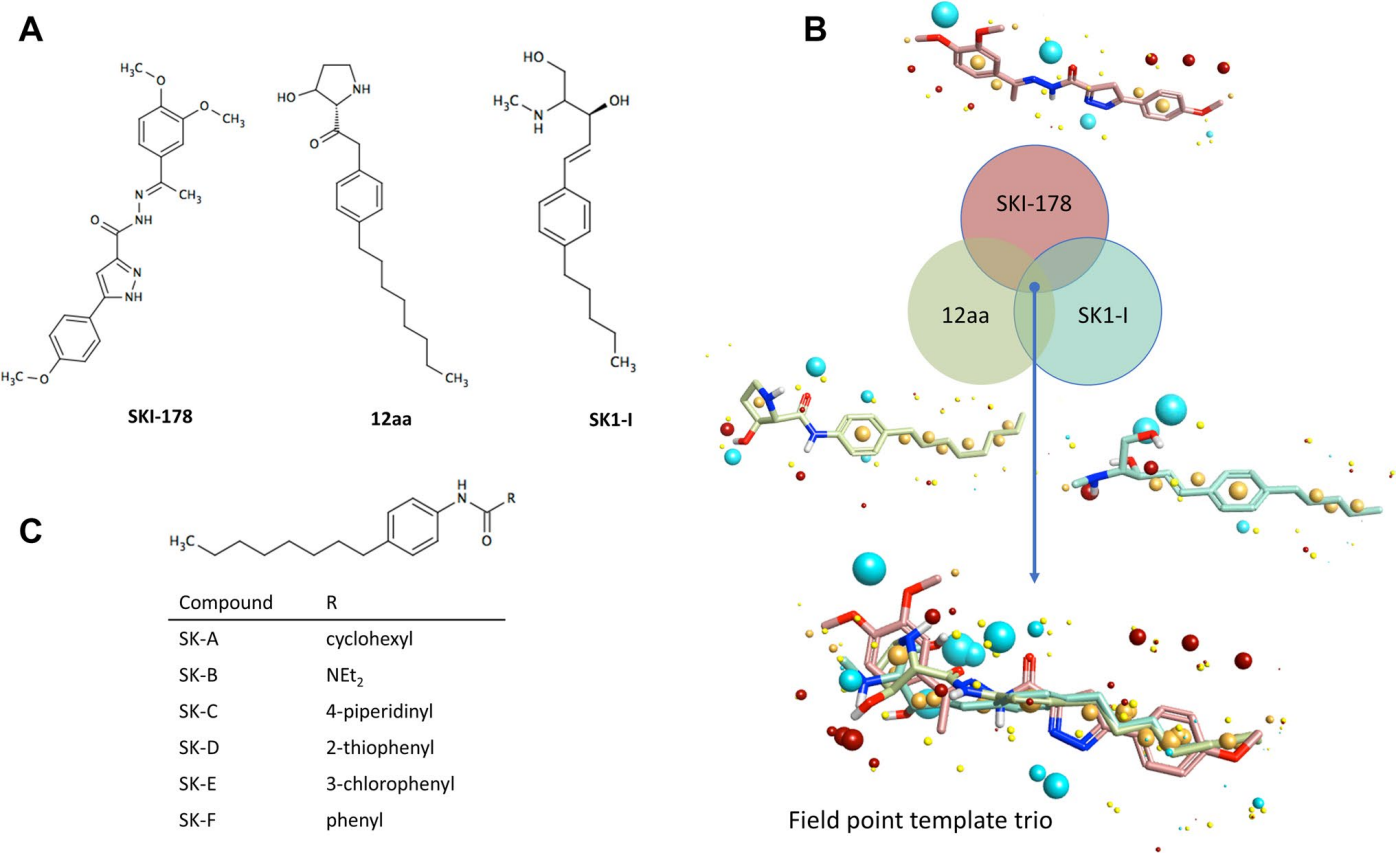
Molecular template design. **a** Structures of three SK1 inhibitors, SKI-178, 12aa and SK1-I, used to prepare field point template pharmacophore. **b** Venn diagram illustrating process used to identify template. FieldTemplater generates a series of conformations that the ligands might adopt at physiological conditions. Field point patterns are calculated for each conformation and molecules compared on this basis. Four types of field point are illustrated: positive (red) and negative (blue) electrostatic points; van der Waals’ (yellow) and surface sticky points (orange). “Duos” are pairs of conformations with high field point similarity, indicated at points where only two circles overlap. A “trio” is a set of three conformations that show high field similarity, indicated at the centre where all three circles overlap with one another. It is likely that such sets represent the bioactive conformations of the SK1 inhibitors, assuming that they all bind to the same protein site. The individual components of the template are shown together with the final assembly. **c** Target structures of potential SK1 inhibitors were identified through “field screening” of available fragment libraries using Spark software. These six compounds were synthesised and tested

The detailed description of the field template method is provided in the supplementary data and was performed as previously described [39]. Briefly, electrostatic and van der Waals field points were calculated for sets of conformations whose energies lay within 5 kcal/mol of the global minimum for the three SK1 inhibitors SKI-178, 12aa and SK1-I (Fig. 1a). This range was chosen on the basis that it covers all conformations likely to exist at physiological temperature (310K). These “molecular field patterns” were compared on a pairwise basis and FieldTemplater used to identify a field pharmacophore, one common field pattern for the three inhibitors (Fig. 1b). At the time of investigation, there was no protein structural information for these particular compounds, and we consider that this ligand-based approach represents the most likely 3D-conformations adopted by the SKI-178, 12aa and SK1-I. Cresset Spark software was then used to find bioisosteric replacements for the portion of the pharmacophore corresponding to the headgroup. The Spark database of 600,000 fragments was originally derived from molecules in ChEMBL and field point patterns calculated for each entity. Virtual libraries were constructed by attaching field point fragments to the 4-*n*-octylphenyl tail of 12aa. The aim is to find bioisosteres with similar shape and electronic properties to the headgroup portion of the field point template pharmacophore. Each compound was overlaid with the field point pharmacophore and high scoring examples i.e. those with the highest field point similarity considered as potential candidates for synthesis. This process led to the design of the six structures of new SK1 inhibitors, SK-A to F. (Fig. 1c).

### Nuclear magnetic resonance spectrometry

Nuclear magnetic resonance (NMR) spectra were recorded in 5-mm tubes calibrated to tetramethylsilane (TMS) at deuterated chloroform (CDCl_3_), on a Bruker AM-400 spectrometer.

### Laboratory synthesis

The detailed description of the compound synthesis is provided in the supplementary data. Compounds **SK-A, SK-B, SK-E, SK-F** (Fig. 1c) were prepared as previously described [40] and compounds **SK-C** and **SK-D** were prepared by amide coupling as previously described [41].

### Cell culture

Cell culture was performed as previously described [42, 43]. Human breast cancer cell line MDA-MB-231 and murine breast cancer cell line 4T1 were purchased from ATCC (Manassas, VA, USA), and maintained in DMEM with 10% fetal calf serum, 50 U/ml penicillin, 50 µg/ml streptomycin, and 2 mM glutamine (Sigma-Aldrich, St. Louis, MO). Cell lines were kept in culture for up to 30 passages. Cells were seeded to reach 70–80% confluence by the end of the treatment.

### Cell treatment and preparation of cell lysates

Cells were plated, serum deprived, and treated as indicated in figure legends. After incubation, cells were washed with ice-cold phosphate-buffered saline (PBS) and harvested.

### Cell viability

Cells were grown in 96-well plates, deprived from serum, and exposed to different treatments as indicated in figure legends. Cellular viability was measured using the 3-(4,5-dimethylthiazol-2-yl)-2,5-diphenyltetrazolium bromide (MTT; 5 mg/ml) colorimetric assay as already described [44].

### RNA extraction, cDNA synthesis and qRT-PCR

Isolation of total RNA from MDA-MB-231 cells was performed using the RNeasy Mini kit (Qiagen, Valencia, CA, USA) as per manufacturer’s instructions. RNA quantity and purity were measured using a NanoDrop ND-1000 Spectrophotometer (Thermo Fisher Scientific, Loughborough, UK). Reverse transcription was performed using precision nanoScript™ reverse transcription kit (PrimerDesign Ltd, Southampton, UK). qRT-PCR was done as already described [26, 44]. Ct values were exported and analysed using qbase software (Biogazelle NV, Zwijnaarde, Belgium).

### Sphingosine kinase assay

SK assay was performed using radiolabeling as previously described [45–47], in conditions favouring SK1 or SK2 activity as required.

### Animal study

Breast cancer allografts were established in 6–8-week-old BALB/c nude mice by injection of 10^6^ 4T1 cells into their mammary pad. Two weeks after implantation, mice were randomised into treatment groups (*n* = 6/group) and treated twice a week for 2 weeks with intraperitoneal injections of dimethyl sulfoxide (DMSO), 5 mg/kg DTX, 5 mg/kg SK-F, 5 mg/kg DTX + 5 mg/kg SK-F. One day after the last treatment, all mice were euthanized and blood was collected. Tumour long and short radii were measured using calipers and tumour volume (v) was calculated using the formula v = 4/3 πab^2^ (a – long radius, b – short radius). Mice and individual organs were weighed and primary tumours were then processed for analysis of SK1 activity as described above. Full blood count and liver function test analysis was done in the Hammersmith hospital biochemical lab.

### Statistical analysis

Data are presented as the mean values of at least three independent experiments normalised to control ± standard error of the mean (SEM) calculated using GraphPad Prism. Statistical significance between two groups was conducted by unpaired Student’s *t* test. *p* value of < 0.05 is considered statistically significant.

## Results

### Design and synthesis of potential SK1 inhibitors

The Field Templater module of Cresset Forge was used to establish the common electrostatic and van der Waals’ features of the three known SK1 inhibitors SKI-178, 12aa and SK1-I (Fig. 1a) (please see detailed description of the method in the supplementary data). Electrostatic and van der Waals’ field points for each conformation of the three SK1 inhibitors were calculated and features of these molecular field patterns common to all three SK1 inhibitors extracted in the form of a field template (Fig. 1b). The final template contains the field points associated with one conformation of each inhibitor and can be considered as a high content field point pharmacophore representing the features responsible for molecular recognition and binding.

New compounds were designed by searching fragment databases for the pattern associated with the polar head group using Cresset Spark. Six structures of new SK1 inhibitors were identified and proposed to be SK1 inhibitors on the basis of the similarity of their field point patterns scored against the pharmcophore template identified above. (Fig. 1c). The compounds were then synthesised and their mass spectra verified (Figures S1–S6).

### Assessment of SK1 inhibitors in cell-free and in vitro settings

**Fig. 2:**
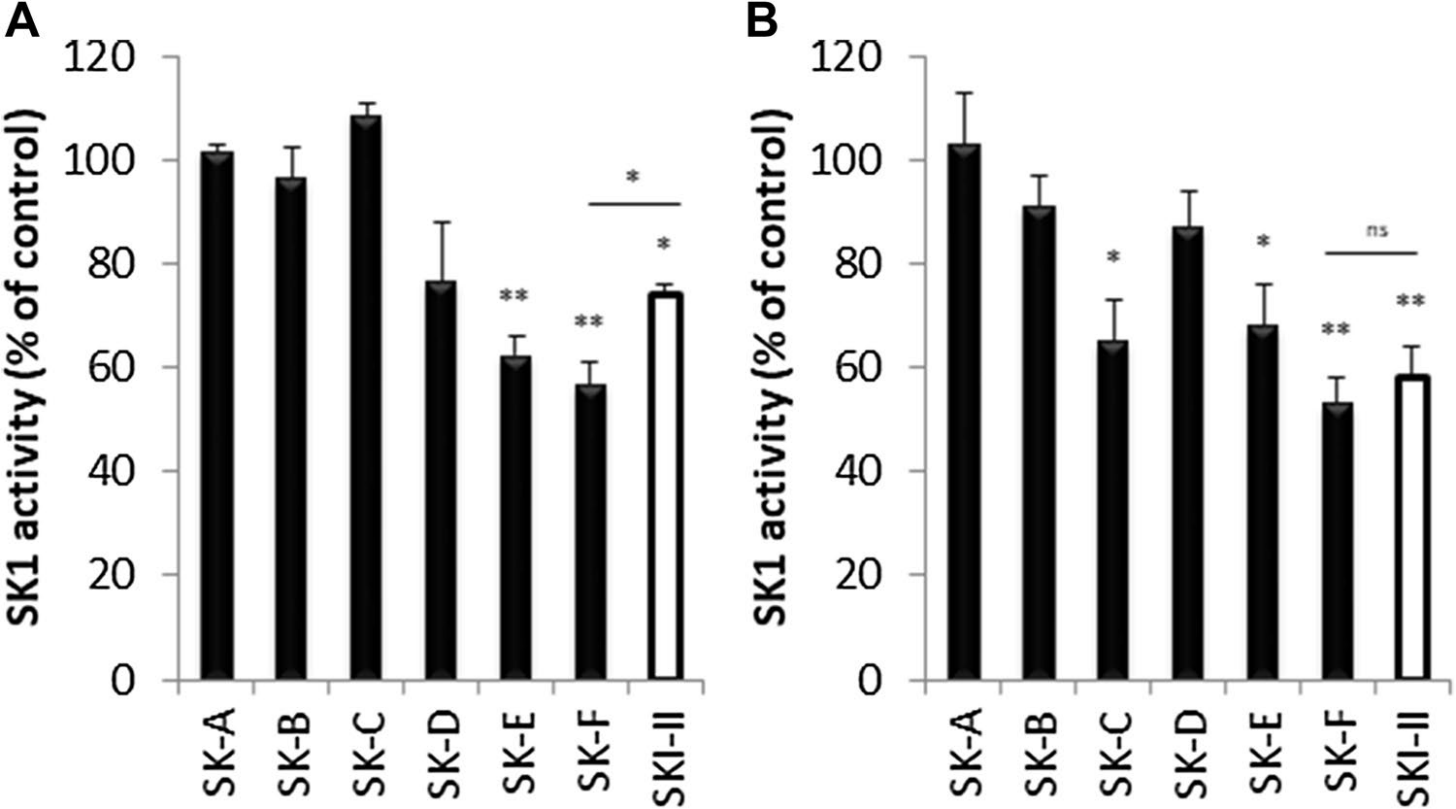
Inhibition of sphingosine kinase 1 in cell-free extract and in vitro. **a** 1 µM of compounds SK-A to SK-F and SKI-II (commercial inhibitor of SK used as a positive control) were incubated in the presence of recombinant SK1 extract (in excess), 1 µM of ATP, and 1 µM exogenous sphingosine for 1 h. **b** MDA-MB-231 cells were incubated with 1 µM of compounds SK-A to SK-F and SKI-II for 6 h. SK1 activity was measured as described in materials and methods. Columns, means of three independent experiments normalised to vehicle; bars, SEM. (*ns*, non-significant; *p < 0.05; **p < 0.01)

The synthesised compounds were then screened for SK1 inhibition. Compounds SK-A to SK-F and SKI-II (commercial inhibitor of SK used as positive control with reported IC_50_ 0.5 µM [14]) at 1 µM were incubated in the presence of recombinant SK1 extract (in excess), 1 µM of ATP, and exogenous 1 µM sphingosine for 1 h followed by measurement of SK1 activity. When compared with SKI-II, SK-F was the most potent inhibitor of SK1 activity in cell-free settings, followed by SK-E and then SK-D (Fig. 2a).

Although the new SK1 inhibitors (SK-D, SK-E and SK-F) demonstrated potency toward inhibition of purified SK1 enzyme, it is important to determine their ability to inhibit endogenous SK1 in an intact cell model. Reportedly, among human tumour cell lines, the breast cancer cell line MDA-MB-231 expresses high levels of SK activity [14]. We treated MDA-MB-231 cells with 1 µM of compounds SK-A – SK-F and SKI-II as the positive control for 6 h and analysed SK1 activity in the cell lysates using SK1 activity assay. SK-C, SK-E and SK-F significantly decreased SK1 activity when compared with vehicle-treated cells (Fig. 2b). Compound SK-F was the most potent inhibitor of SK1 activity in vitro achieving 48.2% inhibition. These results demonstrate that compound SK-F is the most potent inhibitor of SK1 inhibiting not only purified but endogenous SK1 in intact cells (Fig. 2).

**Fig. 3:**
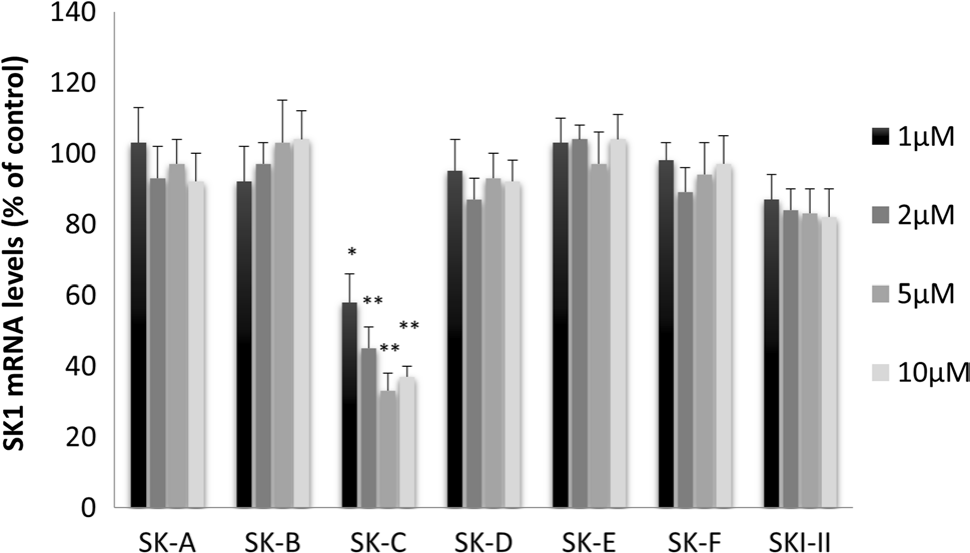
Effect of compounds SK-A to SK-F on SK1 mRNA levels. MDA-MB-231 cells were incubated with 1, 2, 5 and 10 µM of compounds SK-A to SK-F for 24 h. SK1 mRNA expression was measured by qRT-PCR. Columns, means of three independent experiments normalised to control; bars, SEM. (*p < 0.05; **p < 0.01)

Interestingly, compound SK-C was ineffective in cell-free setting, but has significantly inhibited SK1 in vitro. We have therefore evaluated the effect of different concentrations of the new SK1 inhibitors on SK1 mRNA expression in vitro. Except for compound SK-C, the rest of the compounds demonstrated no inhibition of SK1 mRNA expression at any concentration tested (Fig. 3). Overall, of all compounds tested, SK-F demonstrated a sustained SK1 inhibition both in cell-free setting and in vitro without affecting its mRNA expression levels. Dose response analysis of SK1 activity showed that its IC_50_ for purified enzyme was 0.92 µM (Figure S7).

To elucidate whether SK-F is a competitive inhibitor of SK1 and whether it binds to SK2, SK1 and SK2 activities were measured using recombinant SK1 and SK2 with increasing concentrations of sphingosine and the indicated concentrations of SK-F as shown on Lineweaver–Burk plots (Figure S8A, B). SK1 activity was determined in the presence of triton X-100 and SK2 activity was measured in the presence of high salt concentrations, conditions that favour SK1 and SK2, respectively. Our data show that SK-F is a competitive inhibitor of SK1. Km for SK1 was 6.97 ± 1.20 µM, Vmax 98 ± 4 µM/min/mg protein and K_i_ for SK-F 0.46 ± 0.1 µM. Lineweaver–Burk plot for SK2, showed no inhibition of SK2 by SK-F (Figure S8B). Some SK inhibitors such as SKI-II were previously shown to induce proteasomal degradation of SK1 [48, 49]. Our data show that SK-F did not induce any change in SK1 protein (Figure S9).

**Fig. 4:**
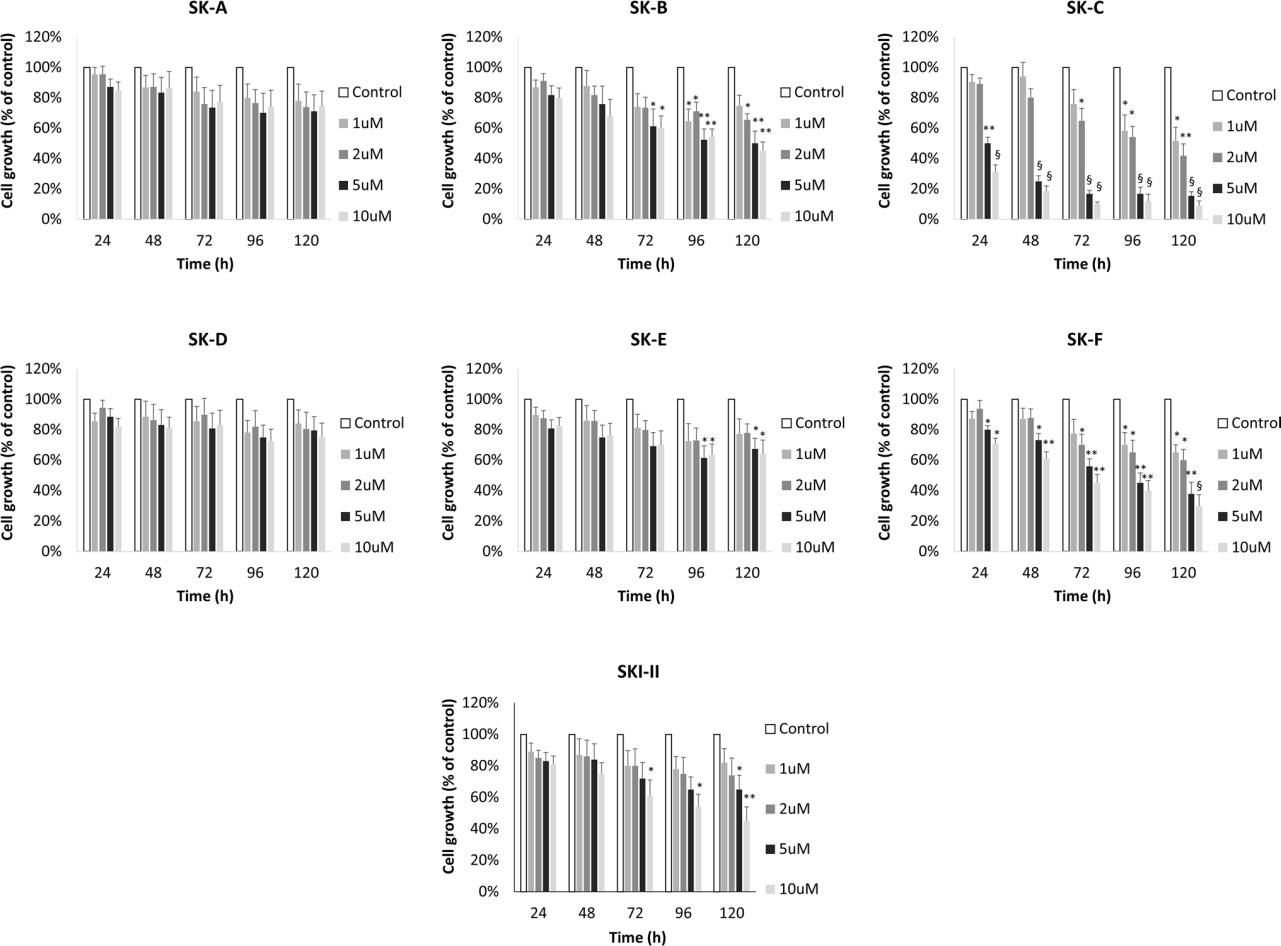
Effect of compounds SK-A to SK-F on breast cancer cell viability. MDA-MB-231 cells were incubated with 1, 2, 5 and 10 µM of compounds SK-A to SK-F for up to 120 h. Cell proliferation was measured using MTT assay. Columns, means of three independent experiments normalised to control; bars, SEM. (*p < 0.05; **p < 0.01; ^§^*p* < 0.001). IC50 levels for SK-F for 72, 96 and 120 h were 7, 4.5 and 3 µM, respectively

It is expected that inhibition or silencing of SK1 will have antiproliferative effect on breast cancer cells [44]. We have assessed the effects of these inhibitors at varying concentrations on triple-negative MDA-MB-231 breast cancer cells using MTT cell proliferation assay. Of all compounds tested, SK-C and SK-F showed the highest effect on MDA-MB-231 cancer cell proliferation (Fig. 4). Based on these data, we have chosen compound SK-F to proceed with for further in vivo testing.

### Compound SK-F effect on SK1 and antitumour activity *in vivo*

To establish the applicability of SK1 inhibitors as anticancer therapeutic agents and for characterization of their whole-body effects, we investigated the in vivo efficacy of compound SK-F. For this purpose, we have used a mouse breast cancer model where triple-negative mouse breast cancer cells 4T1 were injected into the mammary pad of 6–8-week-old female BALB/c nude mice. 4T1 cells have slightly lower levels of SK1 activity than MDA-MB-231 cells and showed a similar response to SK inhibition (Figure S10).

**Fig. 5:**
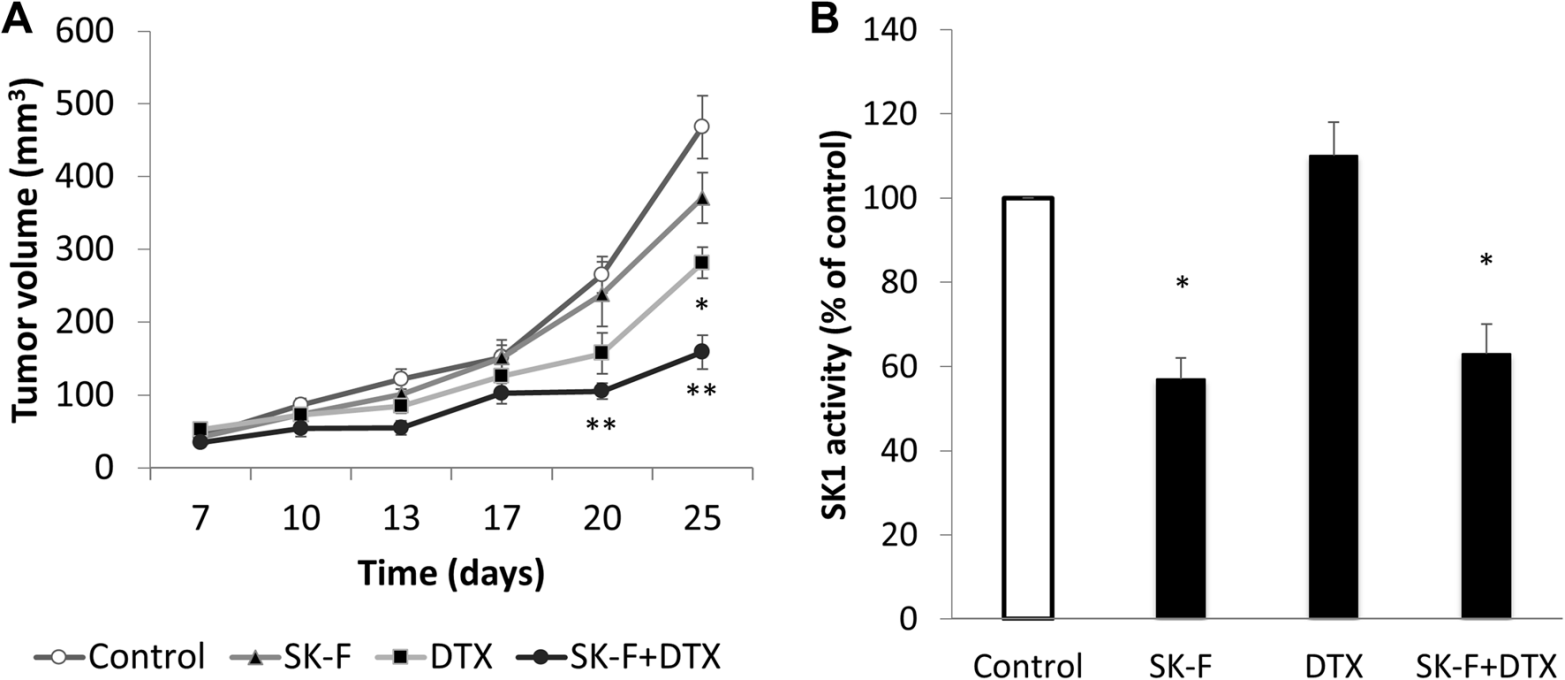
Effect of compound SK-F on breast tumour growth and SK1 activity. Breast cancer allografts were established in BALB/c nude mice by injection of 4T1 cells into their mammary pad. Two weeks after implantation, mice were randomised into treatment groups (*n* = 6/group) and treated twice a week for 2 weeks with intraperitoneal injections of: DMSO, 5 mg/kg DTX, 5 mg/kg SK-F, 5 mg/kg DTX + 5 mg/kg SK-F. **a** Tumour volume in the different treatment groups of 4T1 breast cancer implanted in mouse mammary fat pad. **b** SK1 activity in tumours was measured using radiolabelling. Points, columns, mean of *n* = 6 animals (in B normalised to control); bars, SEM. (*p < 0.05; **p < 0.01)

**Fig. 6:**
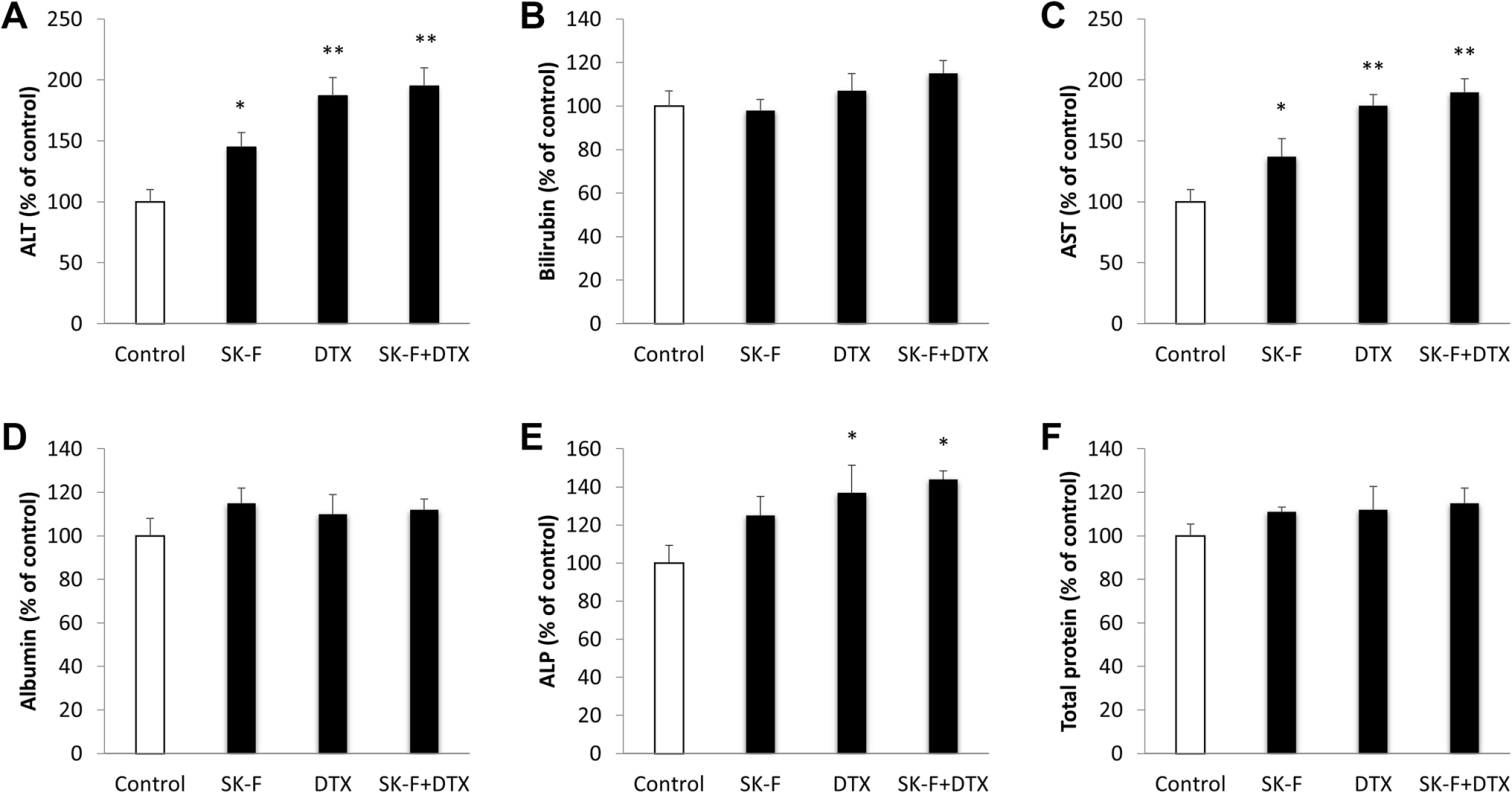
Liver function tests of mice treated with SK-F and docetaxel (DTX). Breast cancer allografts were established in BALB/c nude mice by injection of 4T1 cells into their mammary pad. Two weeks after implantation, mice were randomised into treatment groups (*n* = 6/group) and treated twice a week for two weeks with intraperitoneal injections of: DMSO, 5 mg/kg DTX, 5 mg/kg SK-F, 5 mg/kg DTX + 5 mg/kg SK-F. **a** Alanine transaminases (ALT). **b** Bilirubin. **c** Aspartate transaminase (AST). **d** Albumin. **e** Alkaline phosphatase (ALP). **f** Total protein. Columns, mean of *n* = 6 animals normalised to control; bars, SEM. (*p < 0.05; **p < 0.01)

Tumour-bearing mice were divided into four groups (*n* = 6) and injected with: DMSO, 5 mg/kg DTX, 5 mg/kg SK-F, 5 mg/kg DTX + 5 mg/kg SK-F. Two weeks after the tumour cells inoculation, we began intraperitoneal injections of drug preparations and continued these twice a week for two weeks. Based on our previous studies [25, 26, 50], we have chosen relatively smaller doses of SK-F and DTX in order to investigate their combined effect and potential chemosensitisation of breast tumours to taxane therapies. In the control group, tumours grew progressively and rapidly (Fig. 5a). Treatment with SK-F alone has insignificantly decreased tumour volume relative to control group, with maximal results observed at the end of the study (371 vs 468 mm^3^, respectively). Five mg/kg DTX has induced a significant reduction in tumour volume to 281 mm^3^. The biggest reduction in tumour volume to 159 mm^3^ was observed when DTX was combined with SK-F (Fig. 5a). Similar to in vitro findings, treatment with compound SK-F alone or in combination with DTX, significantly reduced tumour SK1 activity (Fig. 5b). We have further investigated the effects of the used treatments on mouse whole-body toxicity. DTX has reduced body and liver weight, while SK-F had no significant effect **(**Table 1**)**. DTX has induced significant anaemia and leukopenia, while the effects of SK-F were very moderate. In all cases, the effects of combined therapy were similar to DTX alone **(**Table 1**)**. Liver function test was performed as a surrogate marker for chemotherapy-induced liver damage (Fig. 6). DTX induced significant increases in alanine transaminases (ALT) and aspartate transaminase (AST) and a milder increase in alkaline phosphatase (ALP). In comparison to DTX, SK-F had a milder toxicity profile. The combination therapy had similar toxicity profile to DTX alone, while exerting higher antitumour efficacy (Fig. 5a).

**Table 1:** Effect of treatment on whole-body toxicity, (% of control ± SEM)

	Weight of						
	Body	Lungs	Liver	Spleen	Kidney	WCC	Hb
Control	100 ± 7	100 ± 4.8	100 ± 7.5	100 ± 15.4	100 ± 7.1	100 ± 6	100 ± 9
SK-F	91 ± 6^ns^	117.7 ± 5.6*	106.7 ± 5.5^a^	111.2 ± 13.4^a^	118.2 ± 3.6*	78 ± 7*	90 ± 8^a^
DTX	86 ± 7*	99.4 ± 4.a	85.3 ± 11.4^a^	91.3 ± 15.4^a^	97 ± 5.9^a^	54 ± 4^§^	65 ± 7^§^
SK-F + DTX	84 ± 8*	114 ± 7.4*	98.1 ± 3.6^a^	112 ± 10.8^a^	110.8 ± 2.4*	56 ± 6^§^	68 ± 5^§^

^a^ *ns* non-significant

*p < 0.05

**p < 0.01

^§^ *p* < 0.001 in comparison to control

## Discussion

Sphingosine kinase 1 (SK1) is a protooncogenic enzyme expressed in many human tumours and associated with chemoresistance and poor prognosis [3]. It is a potent therapy target and its inhibition chemosensitises solid tumours. Several SK1 inhibitors have been synthesised and tested in biological models with varied success (reviewed in: [51–53]). SK1 crystal structure was not known till recently and previous knowledge was largely based on site-directed mutagenesis and enzyme kinetics (reviewed in: [54]). The most potent SK1 inhibitor PF-543 has a K_i_ of 3.6 nM and an IC_50_ of 2 nM for SK1 [55]. In 2013, Wang et al have reported a crystal structure of SK1, which notably revealed that SK1 3D structure bears no similarity to protein kinases or other lipid kinases and that Asp81 is a key catalytic residue that facilitates direct phosphoryl transfer [56]. A further dynamics study and MM-PBSA binding free energy calculations study showed that residues Ile170, Phe188 and Thr192 in SK1 significantly contribute to a favourable binding energy and specificity for SK1 over SK2 [57]. This has led to a structure-based approach for design and synthesis of a series of SK1 inhibitors [58–60] .

In this study, we have used a ligand-based approach to design six new SK1 inhibitors [39]. If two diverse structures are known to act at the same protein active site, they are presumed to interact in similar ways with the protein. We have used molecular field points to summarise the electrostatic and van der Waals’ properties of molecules. These features can be used as the basis for comparing diverse molecules, particularly in cases where structural similarity is not obvious at first sight. These methods are the basis of the Cresset software packages Forge and Spark which have been successfully applied to scaffold-hopping between different chemotypes [61].

We have used the molecular field patterns of three known SK1 inhibitors: SKI-178, 12aa and SK1-I (Fig. 1a) to establish shared common pharmacophoric features (Fig. 1b). To find the optimal field overlay of two molecules, the field of every conformer of each selected molecule was compared pair-wise until a close field match was found. The conformations from each pair having the most similar fields are assumed to represent the bioactive conformations. The conformations from two molecules often generate many duos with high field similarity. However, the chance that the “common” field is also generated by a third unrelated active molecule affords a useful refinement step. Therefore, by cross correlating all possible duos from three or more molecules acting at the same site, the conformers with the “common” field pattern are more reliably identified as the bioactive conformers.

This 3D information was used as input for Spark (Cresset, UK) in design of new compounds in which the polar headgroup of 12aa was replaced by fragments selected from the Spark database of field point isosteres. A virtual library of potential candidates was assembled and compared to the field point pharmacophore to prioritise examples for synthesis. Six new structures were made (Fig. 1c) and their mass spectra verified (Figures S1–S6).

The synthesised compounds were then screened for cell-free SK1 inhibition. Of six compounds tested only SK-E and SK-F showed statistically significant inhibition of SK1 (Fig. 2a). This result represents a reasonable hit rate for this small set of compounds. Interestingly, three compounds, SK-C, SK-E and SK-F inhibited endogenous SK1 in an intact cell model (Fig. 2b), with compound SK-F achieving 48.2% inhibition, which was superior to SKI-II in our experiment. SK-F did not elicit any changes on SK1 mRNA expression at any concentration tested (Fig. 3).

Some SK inhibitors such as SKI-II were previously shown to induce proteasomal degradation of SK1 [48, 49]. Our data show that only SK-C induced a 75% decrease in SK1 protein (Figure S9). This decrease is, however, similar to the reduction in SK1 mRNA induced by this inhibitor and therefore does not suggest any protein-specific action. Conversely, SKI-II had a minimal effect on SK1 mRNA (Fig. 3), but induced significant decrease in SK1 protein (Figure S9) as previously reported [48, 49]. SKI-178, 12aa and SK1-I have all been shown to be SK1 specific and not to inhibit SK2. SKI-178 was shown to inhibit SK1 at the sphingosine binding site, but not at the ATP binding site [62]. Similarly, SK1-I was shown not to bind to the ATP pocket and to be competitive with the lipid substrate [47]. 12aa has no data regarding its binding site [33]. Lineweaver–Burk plots demonstrate that SK-F is a selective SK1 inhibitor (Figure S8B) and a competitive inhibitor of SK1 (Figure S8A).

It is expected that inhibition or silencing of SK1 will have antiproliferative effect in breast cancer cells [26, 44]. In triple-negative MDA-MB-231 breast cancer cells, SK-F induced significant loss of proliferation (Fig. 4). This is particularly interesting since some recent SK1 inhibitors with sub-micromolar potency (e.g. PF-543) did not demonstrate cytotoxic effects in various cancer cell lines [55, 63, 64]. There is, however, significant evidence showing anticancer cytotoxic effects of SK1 siRNAs [44, 50, 65–67], confirming SK1 as a molecular target for anticancer therapy. SK-C also demonstrated some activity, but as this compound did not show any activity against the isolated enzyme we can conclude that this is either an off-target effect or that this compound is cytotoxic.

Despite the recent determination of the structure of SK1 and a surge in SK1 inhibitor modelling studies, few SK1 inhibitors have been tested in animal cancer models with satisfactory results (reviewed in [68]). To further establish the applicability of SK-F as anticancer therapeutic agent and to characterise its whole-body effects we have investigated its action in the mouse triple-negative breast cancer model. We have combined it with DTX, an antineoplastic taxane that is widely used for the treatment and management of patients with breast cancers. However, drug-related cumulative toxicity, unresponsiveness to DTX therapy, and the development of resistance limit its clinical benefits [69]. In our previous studies, we have shown that 3–5 mg/kg doses of SK1 inhibitor FTY720 sensitise breast tumours to subtherapeutic doses of DTX [25, 26]. Here we have investigated whether SK-F will have a similar effect. Five mg/kg SK-F alone did not significantly alter the tumour volume relative to control group. It has, however, significantly sensitised tumours to 5 mg/kg DTX (tumour volume of 159 mm^3^ in SK-F + DTX group vs 281 mm^3^ in DTX alone) (Fig. 5a). Similar to in vitro findings, treatment with compound SK-F alone or in combination with DTX significantly reduced tumour SK1 activity (Fig. 5b), while DTX alone had no effect. These data are supported by in vitro experiment where 4T1 cells show significant inhibition of SK1 activity in response to SK-F (Figure S10).

For every proposed targeted therapy, it is crucial to identify potential side effects. Our toxicology study showed that contrary to DTX, SK-F did not induce significant mouse body weight loss, or organ weight loss **(**Table 1**)**. SK-F had a milder liver function test profile than DTX and their combination did not have any additive toxicity (Fig. 6). SK-F induced a very mild leukopenia and no anaemia (Table 1). This is a very important finding since the only clinically used SK1 inhibitor FTY720 induces a marked lymphopenia and therefore is not suitable for cancer patients as a free drug. However, in a view of significant DTX toxicity, it may be prudent to consider the use of these drugs in nanoformulations [25, 26].

These findings are particularly important considering a recent report of a safe use of a putative inhibitor of SK safingol in a phase I clinical trial, in combination with cisplatin in 43 cancer patients. Safingol administration did not cause any significant side effects and one patient with adrenal cortical cancer was reported to have a significant regression of liver and lung metastases and another had prolonged stable disease [70]. Overall, through field template screening, we have identified a new SK1 inhibitor SK-F, which demonstrates antitumour activity in vitro and in vivo without overt toxicity when combined with DTX.

## Electronic supplementary material

Below is the link to the electronic supplementary material.

Supplementary material 1 (DOCX 22 KB)

Supplementary material 2 (TIF 1008 KB)

Supplementary material 3 (TIF 857 KB)

Supplementary material 4 (TIF 922 KB)

Supplementary material 5 (TIF 884 KB)

Supplementary material 6 (TIF 827 KB)

Supplementary material 7 (TIF 852 KB)

Supplementary material 8 (TIF 196 KB)

Supplementary material 9 (TIF 601 KB)

Supplementary material 10 (TIF 389 KB)

Supplementary material 11 (TIF 497 KB)
