# Supplementary material for: Field template-based design and biological evaluation of new sphingosine kinase 1 inhibitors
Source: Breast Cancer Res Treat. 2018 Jul 24;172(1):33–43. doi: 10.1007/s10549-018-4900-1 (PMC6208908; doi:10.1007/s10549-018-4900-1)
Supplement: Supplementary file 1 — Supplementary material 1 (DOCX 22 KB) [file 10549_2018_4900_MOESM1_ESM.docx]

**Field templating**

Three previously identified SK1 inhibitors with varied chemical structures: SKI-178 [[1](#_ENREF_1)], 12aa [[2](#_ENREF_2)] and SK1-I [[3](#_ENREF_3)] **(Figure 1A)** were selected as template for establishing a field pattern that could be further developed to identify molecules with potentially improved SK1-inhibiting properties. The field templating method was used, as described by Low and Vinter [[4](#_ENREF_4)].

(a) Hardware and software

The Cresset software packages Forge and Spark ([www.cresset-group.com](http://www.cresset-group.com)) were installed and run on a Mac Pro with 2 x 2.93 GHz 6-Core Intel Xeon processors. Full technical details of both of these packages are available on the Cresset website.

(b) Field template generation and multiconformation field overlay

If two diverse structures are known to act at the same protein active site, they are presumed to interact in similar ways with the protein. In many cases this is clearly independent of their compound class and the field point approach has been developed as a means of summarising features responsible for molecular recognition in terms of electrostatic and van der Waals’ field points. Field points are plotted as points in space around the 3D conformation of the molecule and represent the energetic maxima/minima associated with positive and negative electrostatic regions, hydrophobic regions, and areas of maximum van der Waals attraction. They are calculated in terms of the interaction of appropriate charged and neutral probes at and beyond the molecular surface and coloured to reflect positive (red) or negative (blue) charge; van der Waals (yellow) and surface sticky points (orange). To find the optimal field overlay of two molecules, the field of every conformer of each selected molecule is compared pair-wise until a close field match is found. The conformations from each pair having the most similar fields are assumed to represent the bioactive conformations. Pairs of molecules found to have a “common” field are termed “duos”. Conformers with the “common” field were identified by cross correlating all possible duos from three molecules acting at the same site (**Venn diagram in Figure 1B**). Combining the “common” fields of progressively more diverse active molecules should increase the definition of the binding site requirements, and in so doing form a “field template” for that binding site which can be regarded as a high definition field point pharmacophore. The FieldTemplater module of Forge was used to create an SK1-receptor field template from three active inhibitors SKI-178, 12aa, and SK1-I (**Figure 1A**). Notably, a common conformation of 12aa was indicated in each of the “trio” templates. This conformation was used as a basis to design novel compounds.

(c) Design of new SK1 inhibitors

The strategy was to replace the polar head group with bioisosteres suggested by the Cresset package Spark. This contains a database of field point patterns that reflect fragments of molecules that are commonly represented in the structures in the ChEMBL database (https://www.ebi.ac.uk/chembl/). Virtual libraries of compounds were constructed and examples selected for synthesis on the basis of their field similarity scores generated from overlay with the Field point template. This led to identification of compounds SK-A to SK-F as candidates for synthesis (**Figure 1C**)

###

### Laboratory synthesis

Analysis

NMR spectra **(Figures S1-S6)** were recorded in 5 mm tubes calibrated to tetramethylsilane (TMS) at deuterated chloroform (CDCl_3_), on a Bruker AM-400 spectrometer. 2D ^1^H and ^13^C NMR experiments were carried out where appropriate to aid assignment of spectra. Analytical RP-HPLC experiments were performed on a Gilson analytical RP-HPLC equipped with an autosampler using a methanol gradient in water of 50–100% over 30 min. Samples were characterised by electrospray ionisation (ESI) (positive reflectron mode) mass spectrometry, recorded on a Micromass Autospec-Q spectrometer (of note the compound dimers are present on the spectra due to high compound concentration)

Compounds **SK-A, SK-B, SK-E,** and **SK-F** **(Figure 1C)** were prepared as described by [[5](#_ENREF_5)]. Briefly, the acid chloride (0.10 mmol, 1 eqv.) was added dropwise to a solution of 4-octylaniline (0.223 mL, 0.974 mmol, 1.1 eqvs.) and triethylamine (0.25 mL, 2 mmol) in dichloromethane (5 mL) and stirred at room temperature for 4h throughout which the reaction was observed by TLC at 30-45 minute intervals. Distilled H_2_O was added and the mixture extracted with dichloromethane (20 mL) three times. The combined extracted organic layers were dried over magnesium sulphate, filtered and the solvent was evaporated under reduced pressure. The crude product was purified by column chromatography.

**Compound SK-A.** Product isolated as a white crystalline solid (159 mg, 56% yield). R*_f_*  = 0.27 (EtOAc–n-hexane, 1:20); *δ*_H_/ppm (400 MHz, CDCl_3_) 7.42 (2H, d, Ar-H), 7.13 (2H, d, Ar-H), 7.06 (1H, br s, N-H), 2.56 (2H, t), 2.22 (1H, tt), 1.90 (5H, dd), 1.74-1.67 (4H, m), 1.37-1.20 (14H, m), 0.88 (3H, t) ; *m*/*z* (ES), 316 ([M + H]^+^); HRMS, found 316.2627.

**Compound SK-B.** Product isolated as a brown oil (152 mg, 55% yield). R*_f_*  = 0.13 (EtOAc–n-hexane, 1:15); *δ*_H_/ppm (400 MHz, CDCl_3_) 7.29 (2H, d, Ar-H), 7.10 (2H, d, Ar-H), 6.19 (1H, br s, N-H), 3.38 (4H, q), 2.55 (2H, t), 1.38-1.19 (16H, m), 0.88 (3H, t); *m*/*z* (ES), 305 ([M + H]^+^).

**Compound SK-E.** Product isolated as a white crystalline solid (162.9 mg, 53% yield). R*_f_*  = 0.30 (EtOAc–n-hexane, 1:9); *δ*_H_/ppm (400 MHz, CDCl_3_) 7.85 (1H, t, Ar-H), 7.75 (1H, dd, Ar-H), 7.69 (1H, s, Ar-H), 7.53 (2H, d, Ar-H), 7.44 (1H, t, Ar-H), 7.20 (2H, d, Ar-H), 2.61 (2H, t), 1.31-1.27 (10H, m), 0.89 (3H, t); *m*/*z* (ES), 344 ([M + H]^+^).

**Compound SK-F.** Product isolated as a white crystalline solid (189.7 mg, 68% yield). R*_f_*  = 0.33 (EtOAc–n-hexane, 1:9); *δ*_H_/ppm (400 MHz, CDCl_3_) 7.88 (2H, dd, Ar-H), 7.58-7.48 (5H, m, Ar-H), 7.20 (2H, d, Ar-H), 2.60 (2H, t), 1.66-1.58 (3H, m), 1.37-1.21 (11H, m), 0.89 (3H, t); *m*/*z* (ES), 309 ([M + H]^+^).

Compounds **SK-C** and **SK-D** were prepared by amide coupling as described by [[6](#_ENREF_6)]. Briefly, a solution of N’,N’-dicyclohexylcarbodiimide (147.3 mg, 0.714 mmol), and 1-hydroxybenzotriazole (96.5 mg, 0.714 mmol) in dichloromethane was added to the solution of acid (1 eqv.) in dichloromethane. 4-octylaniline (0.223 mL, 0.975 mmol, 1.1 equivalents) in dichloromethane was then added and the resulting mixture was stirred at room temperature (25 °C) for 24h, checking by TLC at intervals throughout. A saturated ammonium chloride solution was added and the solution extracted three times with dichloromethane (20 mL). The combined extracted organic layers were dried over magnesium sulphate, filtered and the solvent was evaporated under reduced pressure.

Compound **SK-C**. The Boc-protecting group present on the heterocyclic nitrogen in the acid reagent was removed by stirring with trifluroacetic (TFA) acid (2 mL, 0.026mol) in DCM (4ml) for 10 minutes at room temperature. All volatile components were then removed by evaporation at reduced pressure and the compound was purified by HPLC to give a white powdery solid. (2.5 mg, 1% yield).

Given the low yield of this compound, the following characterisation data corresponds to the purified compound before deprotection: R*_f_*  = 0.20 (MeOH–DCM, 1:25); *δ*_H_/ppm 7.41 (2H, d, Ar-H), 7.14 (2H, d, Ar-H), 7.09 (1H, br s N-H), 4.27-4.11 (3H, m), 4.02 (2H, d), 3.55–3.42 (3H, m), 2.87-2.73 (2H, m), 2.57 (2H, t), 2.37 (1H, tt), 1.98-1.86 (6H, m), 1.81-1.59 (9H, m), 1.48 (8H, s), 1.42-1.23 (15H, m), 1.22-1.05 (7H, m), 0.88 (3H, t); *m*/*z* (ES), 317 ([M + H]^+^).

Compound **SK-D**. Purification by column chromatography failed to remove the aniline starting reagent. The purified compound was thus extracted in 2 M HCl_(aq)_ and DCM (20 ml) three times to precipitate out the aniline impurity as a salt into aqueous phase. The purified compound present in the organic layer was retained by evaporating off organic solvent at reduced pressure to give a gold-brown solid. (102 mg, 50% yield). Rf = 0.75 (DCM only); *δ*_H_/ppm 1.64 (2H, d, Ar-H), 7.57 (1H, dd, Ar-H), 7.54 (2H, dd, Ar-H), 7.21 (2H, d, Ar-H), 7.16 (1H, dd, Ar-H), 2.62 (2H, t), 1.69-1.60 (5H, m) 1.39– 1.24 (14H, m), 0.91 (3H, t) ; *m*/*z* (ES), 316 ([M + H]^+^).

**Supplementary references**

1. Hengst, J., et al., *Development of a Sphingosine Kinase 1 Specific Small-Molecule Inhibitor.* Bioorganic & Medicinal Chemistry Letters, 2010.

2. Xiang, Y.B., et al., *Discovery of novel sphingosine kinase-1 inhibitors. Part 2.* Bioorganic & Medicinal Chemistry Letters, 2010. **20**(15): p. 4550-4554.

3. French, K.J., et al., *Antitumor activity of sphingosine kinase inhibitors.* J Pharmacol Exp Ther, 2006. **318**(2): p. 596-603.

4. Low, C.M.R. and J. Vinter, *Rationalizing the activities of diverse cholecystokinin 2 receptor antagonists using molecular field points.* Journal of medicinal chemistry, 2008. **51**(3): p. 565-573.

5. Itsenko, O., T. Kihlberg, and B. Långström, *Photoinitiated Carbonylation with [11C]Carbon Monoxide Using Amines and Alkyl Iodides.* The Journal of Organic Chemistry, 2004. **69**(13): p. 4356-4360.

6. Chan, L.C. and B.G. Cox, *Kinetics of amide formation through Carbodiimide/N-Hydroxybenzotriazole (HOBt) couplings.* Journal of Organic Chemistry, 2007. **72**(23): p. 8863-8869.
