# Supplementary figures and images for: Field template-based design and biological evaluation of new sphingosine kinase 1 inhibitors
Source: Breast Cancer Res Treat. 2018 Jul 24;172(1):33–43. doi: 10.1007/s10549-018-4900-1 (PMC6208908; doi:10.1007/s10549-018-4900-1)

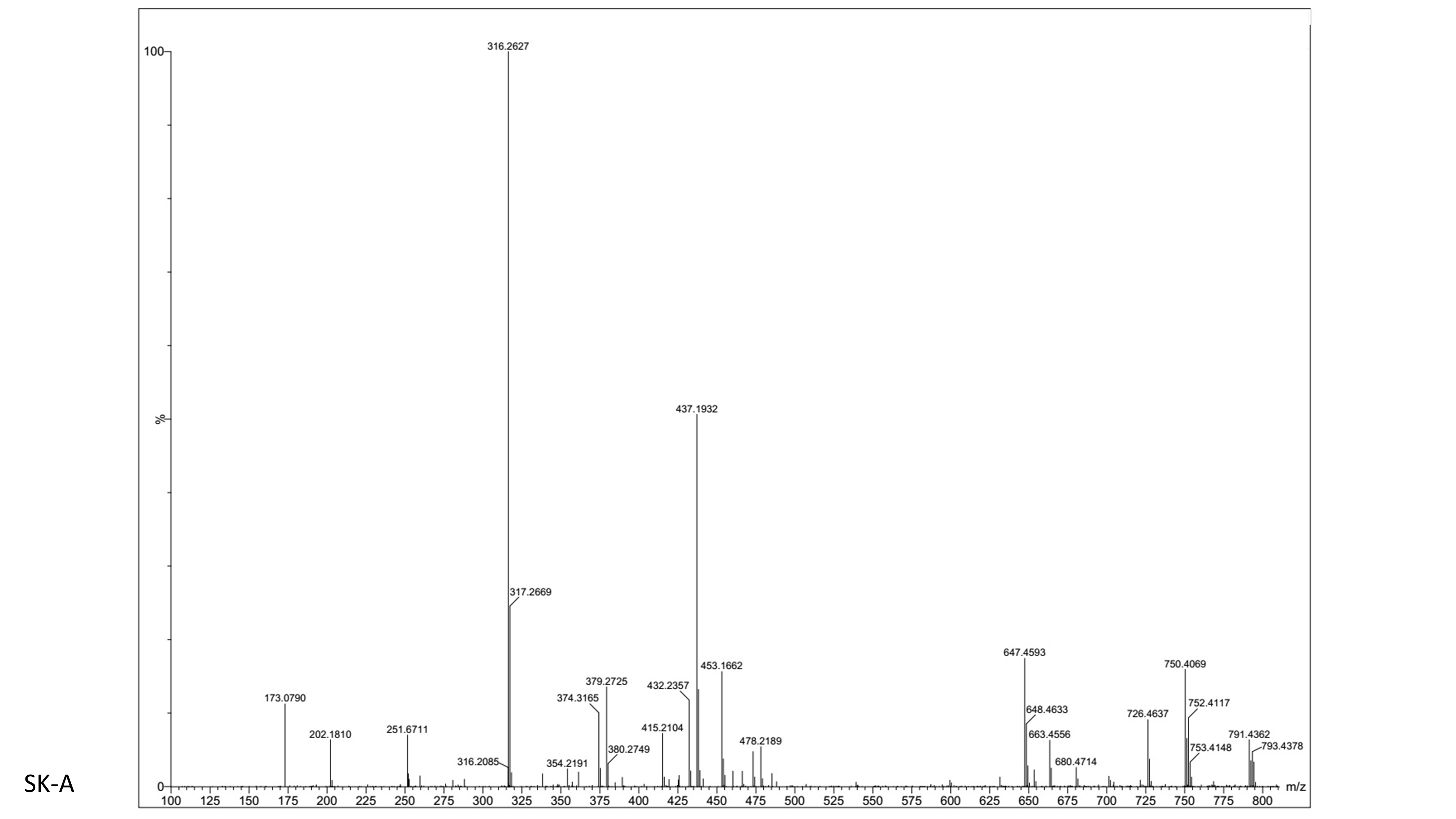

Supplement: Supplementary file 2 — Supplementary material 2 (TIF 1008 KB) [file 10549_2018_4900_MOESM2_ESM.tif]

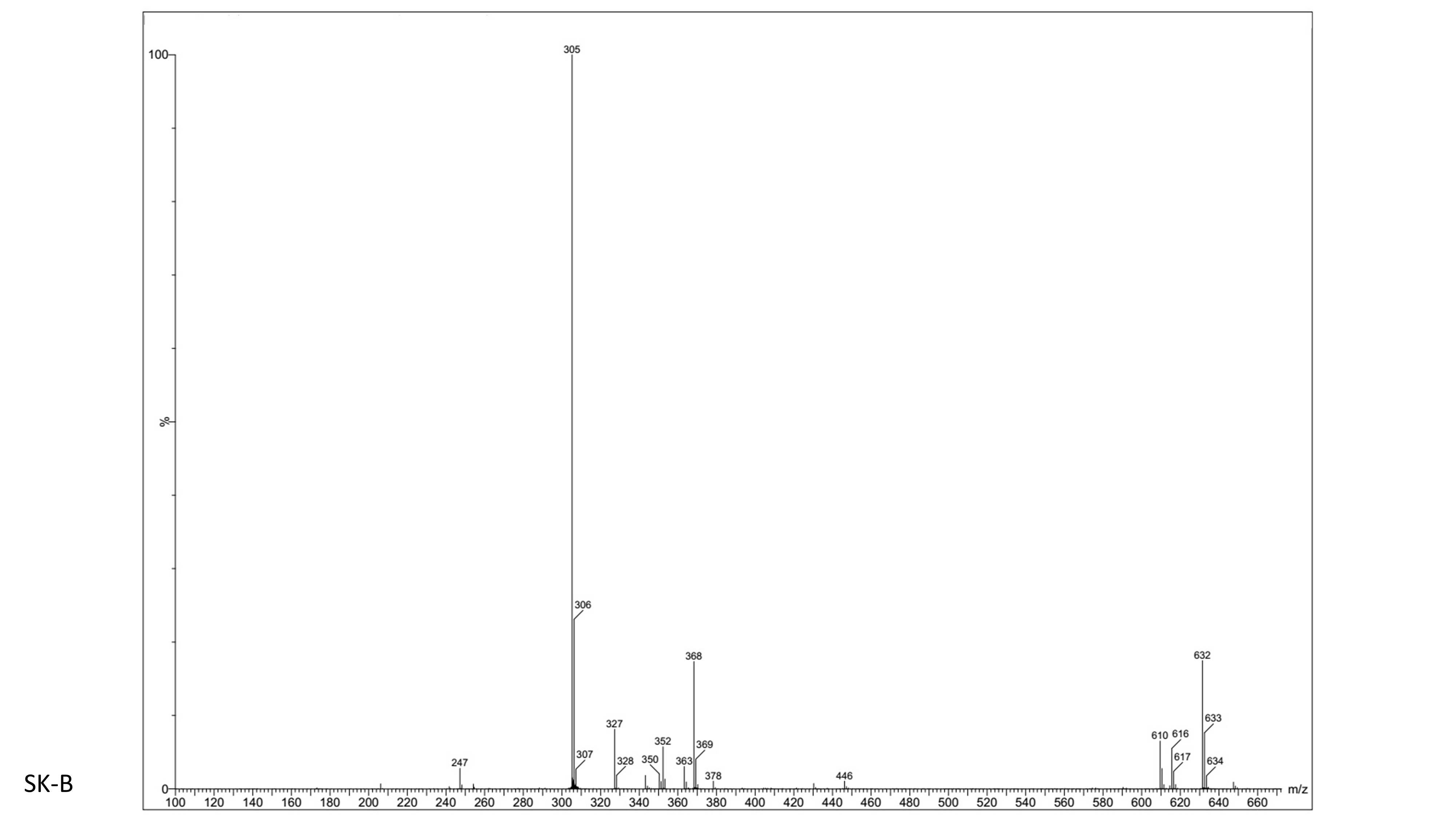

Supplement: Supplementary file 3 — Supplementary material 3 (TIF 857 KB) [file 10549_2018_4900_MOESM3_ESM.tif]

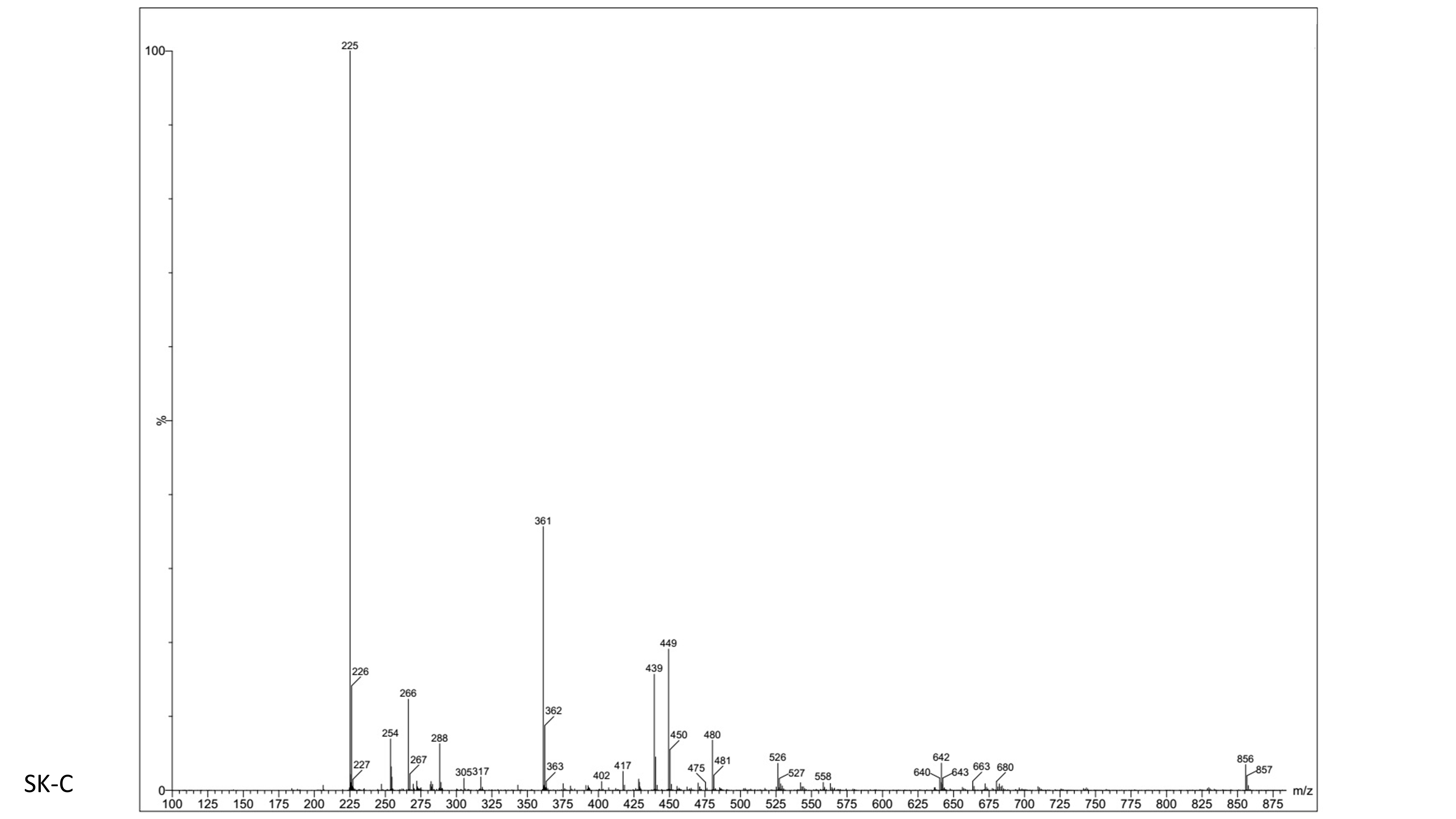

Supplement: Supplementary file 4 — Supplementary material 4 (TIF 922 KB) [file 10549_2018_4900_MOESM4_ESM.tif]

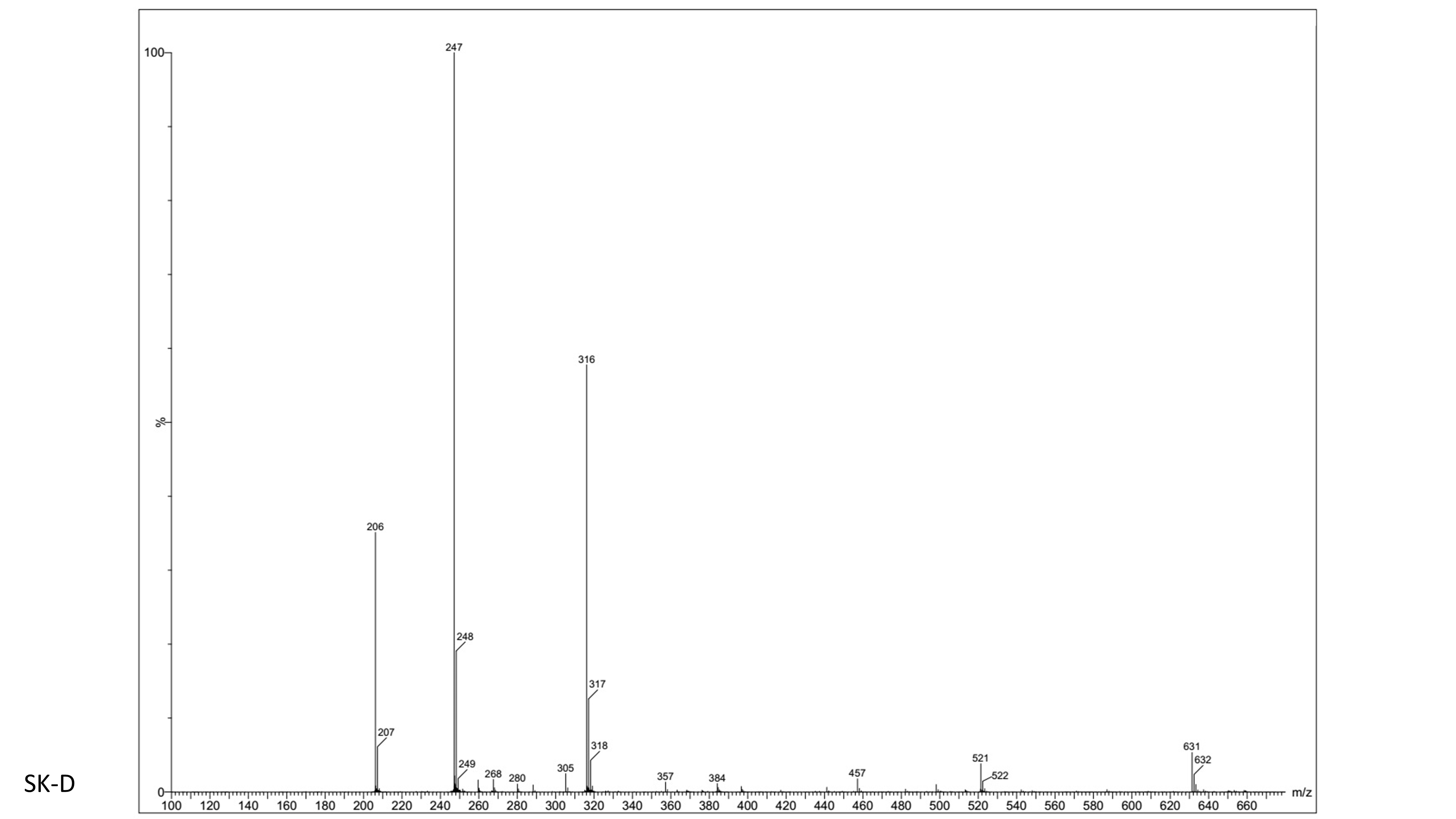

Supplement: Supplementary file 5 — Supplementary material 5 (TIF 884 KB) [file 10549_2018_4900_MOESM5_ESM.tif]

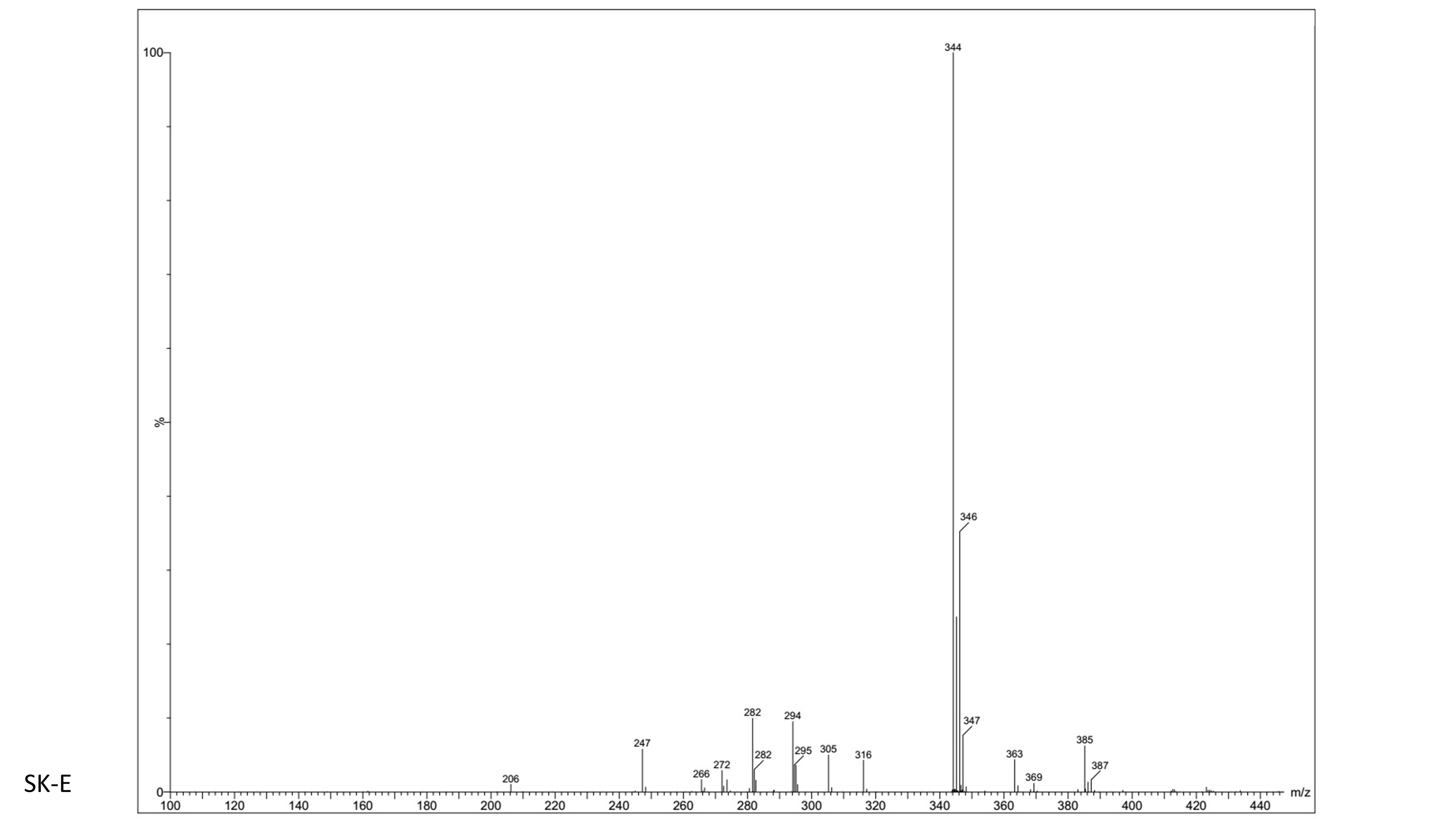

Supplement: Supplementary file 6 — Supplementary material 6 (TIF 827 KB) [file 10549_2018_4900_MOESM6_ESM.tif]

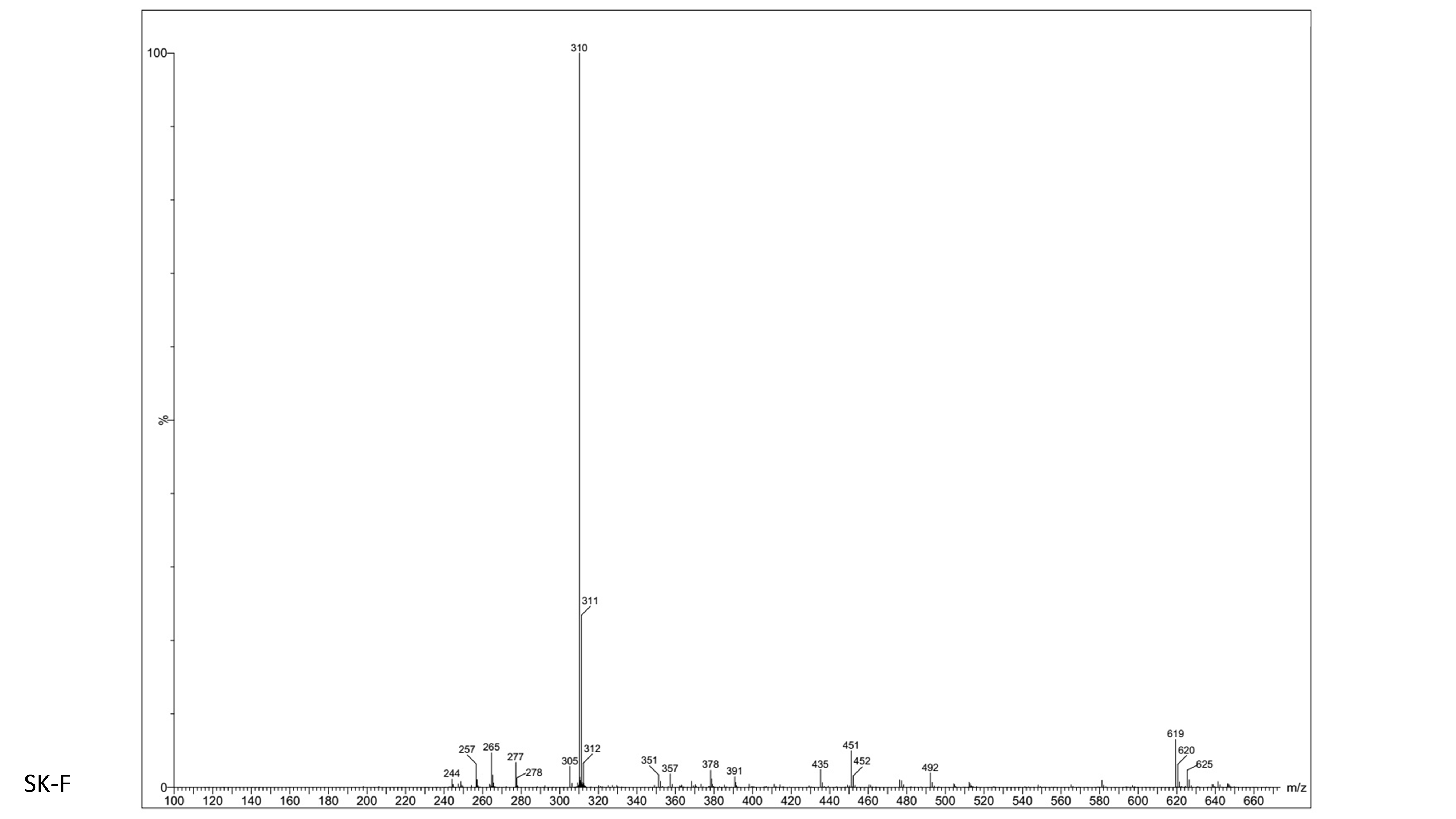

Supplement: Supplementary file 7 — Supplementary material 7 (TIF 852 KB) [file 10549_2018_4900_MOESM7_ESM.tif]

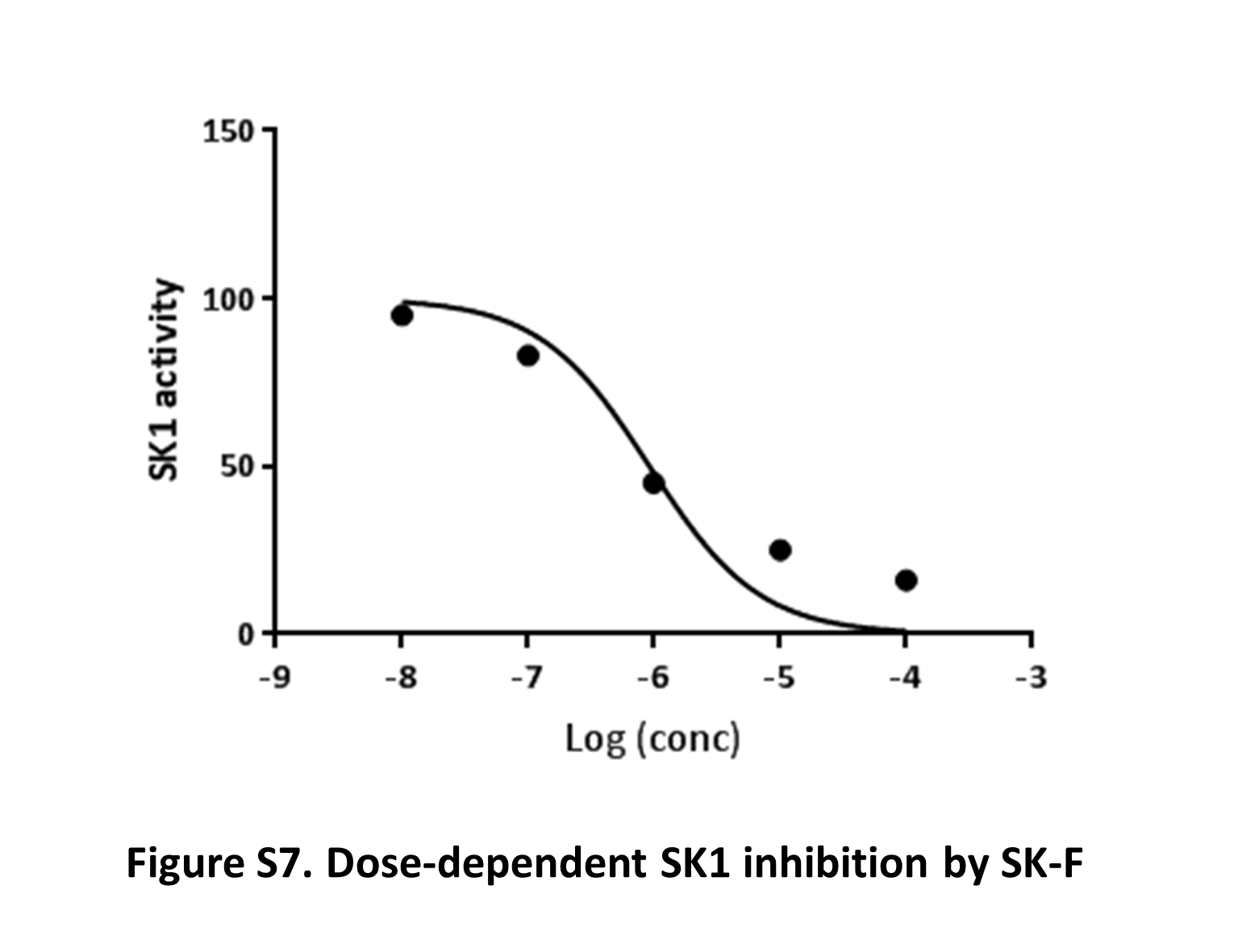

Supplement: Supplementary file 8 — Supplementary material 8 (TIF 196 KB) [file 10549_2018_4900_MOESM8_ESM.tif]

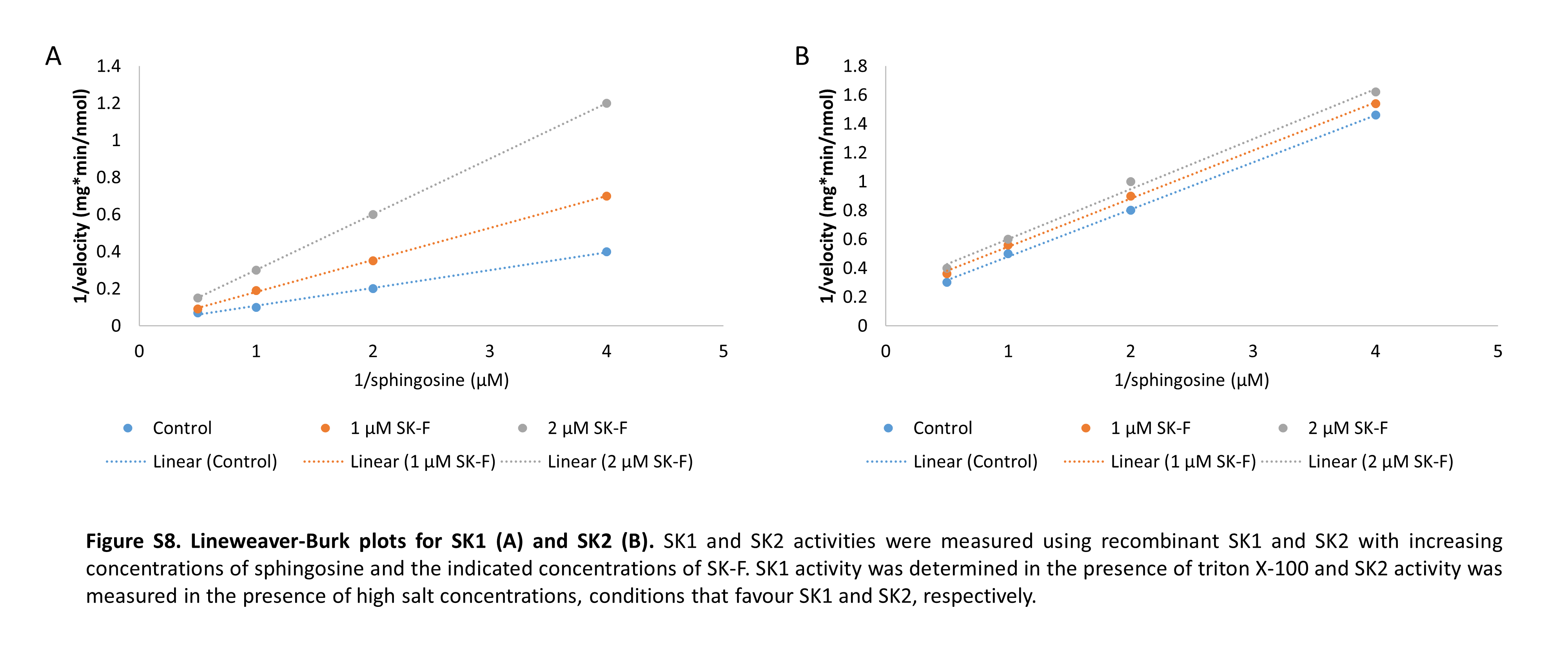

Supplement: Supplementary file 9 — Supplementary material 9 (TIF 601 KB) [file 10549_2018_4900_MOESM9_ESM.tif]

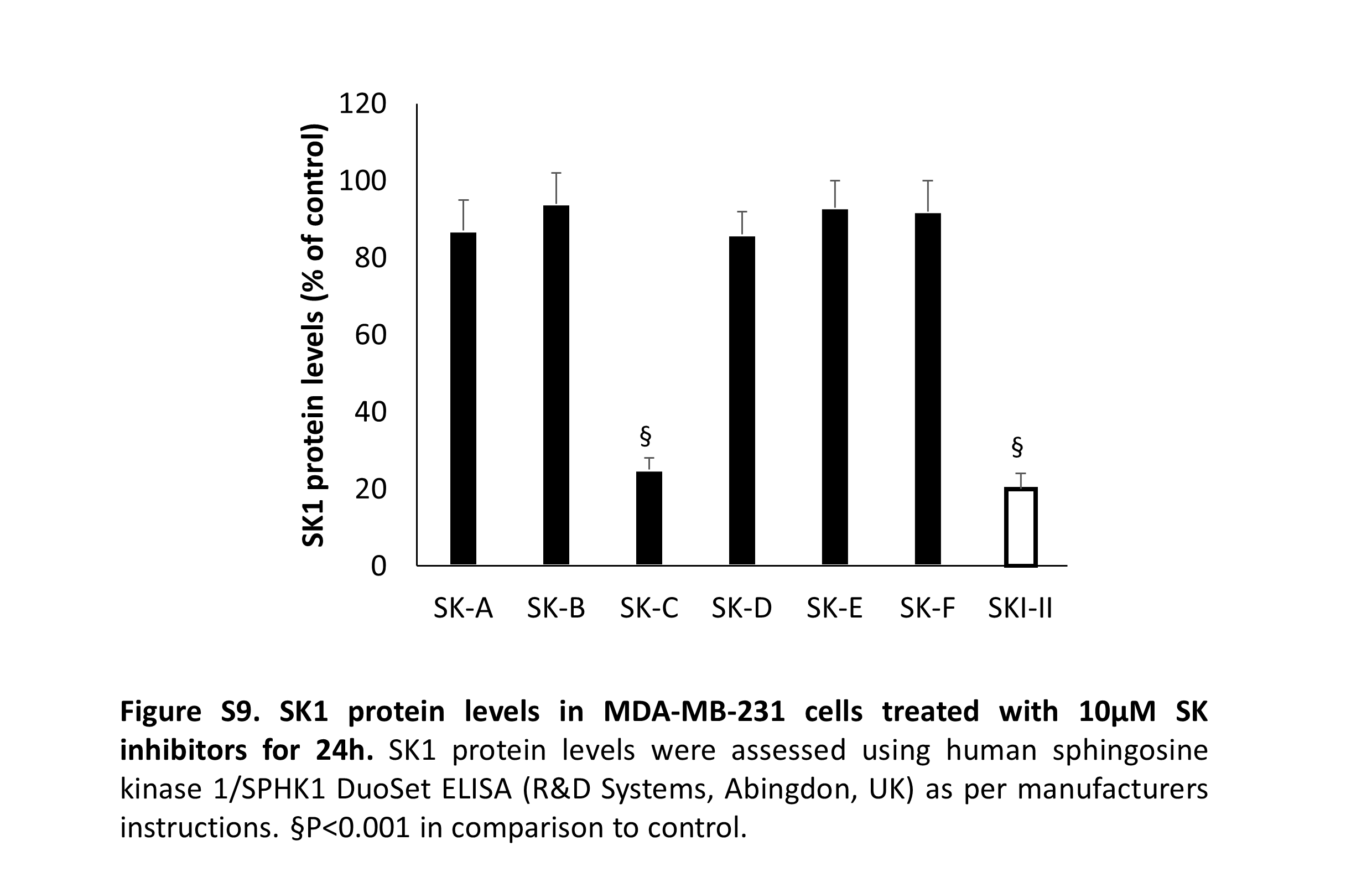

Supplement: Supplementary file 10 — Supplementary material 10 (TIF 389 KB) [file 10549_2018_4900_MOESM10_ESM.tif]

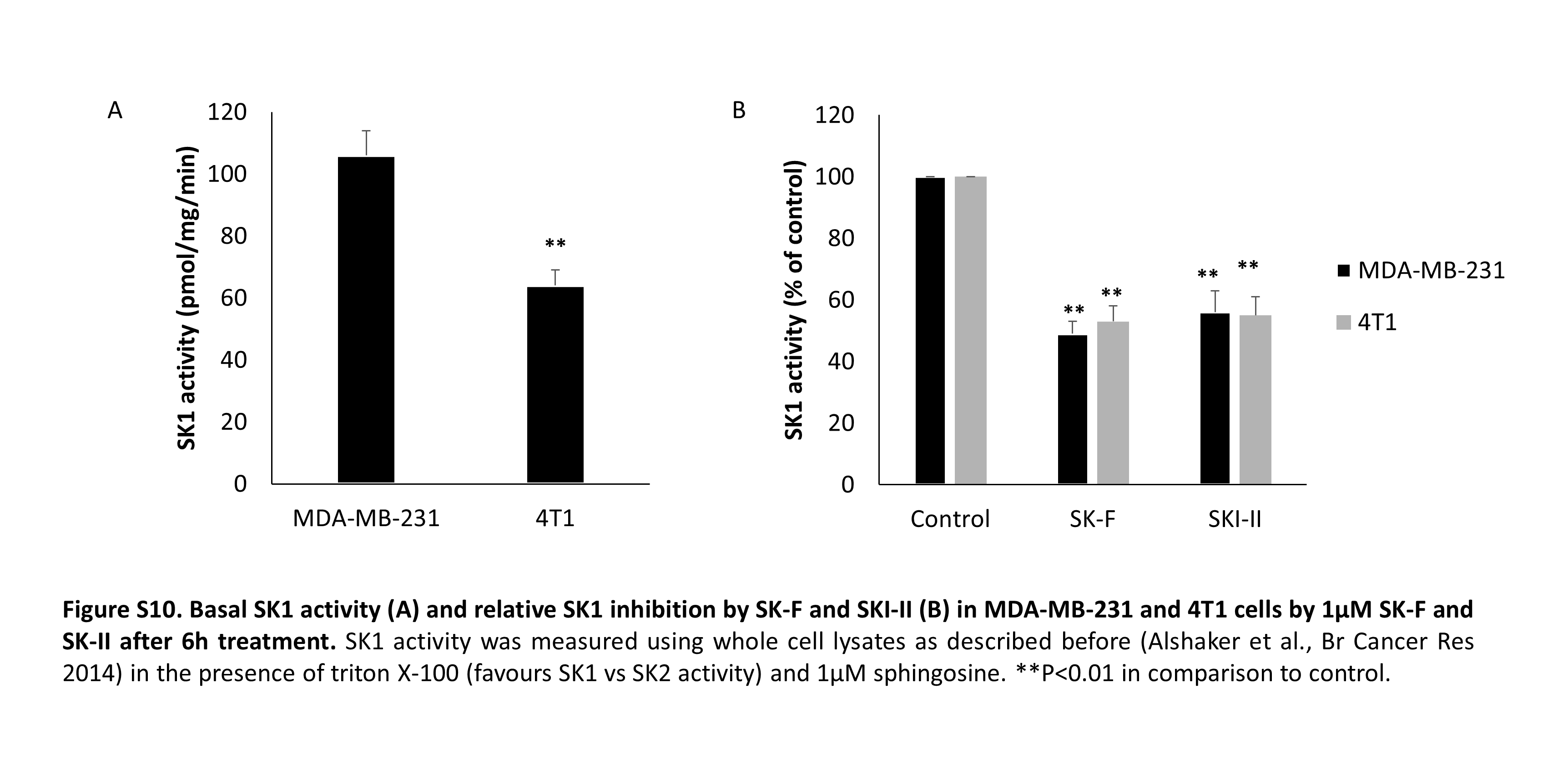

Supplement: Supplementary file 11 — Supplementary material 11 (TIF 497 KB) [file 10549_2018_4900_MOESM11_ESM.tif]
